# Seedling Establishment of *Dendrophylax lindenii* Reintroduced In Situ: Implications for Conservation and Management of a Leafless Epiphytic Orchid

**DOI:** 10.3390/plants15060858

**Published:** 2026-03-10

**Authors:** Adam R. Herdman, Michael E. Kane, Ernesto B. Mújica, Mark W. Danaher, Lawrence W. Zettler, Paulina Quijia-Lamiña, Héctor E. Pérez, Carrie Reinhardt-Adams

**Affiliations:** 1 Horticulture Sciences Department, University of Florida Institute of Food and Agricultural Sciences, Gainesville, FL 32603, USA; micropro@ufl.edu (M.E.K.); heperez@ufl.edu (H.E.P.); rein0050@ufl.edu (C.R.-A.); 2 Orquideario Soroa, Carretera a Soroa Km. 8, Candelaria, ART, Cuba; ernestom.orchid@gmail.com; 3 Florida Panther National Wildlife Refuge, U.S. Fish & Wildlife Service, Immokalee, FL 34142, USA; mark_danaher@fws.gov; 4 Department of Biology, Illinois College, 1101 W College Ave., Jacksonville, IL 62650, USA; lwzettle@ic.edu; 5 Microbiology & Plant Pathology Department, University of California Riverside, 900 University Ave., Riverside, CA 92521, USA; paulina.quijialamina@ucr.edu

**Keywords:** bryophyte facilitation, Kaplan–Meier survival analysis, leafless epiphyte, long-term monitoring, microsite selection, neighbor effects, plant reintroduction, restoration ecology, root attachment

## Abstract

Reintroduction is increasingly used to support declining plant species, particularly epi-phytic orchids that display complex ecological requirements. We evaluated the seven-year performance of 123 asymbiotically propagated ghost orchid (*Dendrophylax lindenii*) seedlings that were reintroduced into a natural pond-apple/pop ash slough on the Florida Panther National Wildlife Refuge. Annual monitoring of this leafless epiphytic species assessed survival, root attachment, and reproduction, with respect to host tree bark texture, host tree species, and neighboring epiphytes. Eighteen individuals (15%) persisted after 83 months, and median survival time was 47 months. Reintroduced orchids near ferns experienced 2–4-fold higher mortality compared with those near mosses, lichens, or other ghost orchids, while survival exceeded 36% at 71 months for individuals placed adjacent to bryophytes. Despite flowering in up to 19% of surviving individuals, no seed capsule reached maturity, indicating that sexual reproduction remains a major bottleneck for population persistence. However, low reproductive output and gradual attrition suggest that reintroduction alone is unlikely to produce self-sustaining populations without addressing the likely genetic constraints, the possible pollinator service constraints, and microsite drivers of persistence. This study highlights the importance of extended monitoring and microsite selection strategies for leafless epiphytic orchid conservation.

## 1. Introduction

The ongoing rapid decline of global biodiversity has sparked an urgent need to preserve and maintain species faced with imminent extinction, especially in forested tropical habitats. Trees in these habitats support an abundance of non-woody epiphytic plant communities that may consist of bryophytes, ferns, bromeliads, and orchids. These epiphytes tend to colonize trunks, branches, and twigs where sunlight is more plentiful higher in the canopy. Of these, orchids represent one of the largest plant families (Orchidaceae) with 30,381 species in 703 genera [1,2].

Orchids are among the most vulnerable of all plants despite their diversity and global distribution, due in part to their sensitivity to environmental changes [3,4]. These changes can negatively affect existing orchid populations and the cohabitating organisms critical to their persistence (e.g., insect pollinators, mycorrhizal fungi). This may be why orchids are among the first plants to disappear from the landscape [1]. Additionally, IUCN assessments are not conducted on a random sample of orchid species but often a targeted narrowly distributed species, endemics or potentially threatened taxa that are being assessed first. Thus, the proportion of threatened species among assessed taxa may not be representative of orchids overall. Most deleterious changes are directly linked to human activities such as deforestation [1,5], the spread of non-native invasive species [6], and fluctuating weather patterns attributed to increasing carbon emissions [7]. Given their widespread appeal, poaching of critically rare showy species from protected habitats remains a threat that gradually erodes genetic diversity. As a result, nearly half of all orchid species assessed are now either threatened or endangered [1], and this number is expected to rise this century [5]. Mitigating this trend will depend on how effectively these threats are minimized and coupled with conservation efforts that restore the rarest and least-studied species on the brink of extinction [1].

The majority (69%) of orchid species exist as tropical epiphytes [8,9]. Epiphytic orchids occupy a wide range of ecological niches leading to diverse floral architecture [10] influenced by a diverse mycorrhizal fungi community on host trees [11,12]. The presence of these fungi influences fine-scale distribution by facilitating where on the tree orchid seeds are able to germinate. Likewise, fungi may also contribute to orchid carbon acquisition via mycotropy, augmenting carbon gained through photosynthetic leaves and roots.

Florida is home to about 100 native orchid species, making it the most orchid-rich states in the U.S. [13]. Roughly half occur only in the subtropical south, especially in the Fakahatchee Strand, an 8 km × 30 km stretch of swamp and slough habitat [14,15]. This area supports many native epiphytic orchids, most of which have declined due to habitat loss, altered hydrology, and climate change [16]. The ghost orchid has become a conservation priority due to its rarity, poaching risk, and sensitivity to environmental change. Poaching, including high-profile cases like those described in The Orchid Thief [17], continue to threaten wild populations despite propagation protection efforts.

Leafless epiphytic orchids, which comprise only ca. 300 species worldwide, may rely on mycotrophy to a greater extent than their leaf-bearing counterparts to overcompensate for carbon they would otherwise gain by having photosynthetic leaves [18]. These orchids photosynthesize using an extensive root system harboring chloroplasts and stomatal complexes within the cortical region [19]. These roots also contain mycorrhizal fungi that supply a source of water in addition to carbon following their digestion [20]. Many of these leafless epiphytes are represented in Asia by three genera (*Chiloschista*, *Phalaenopsis*, *Taeniophyllum*). In the New World, specifically within the Greater Antilles, Central America and southern Mexico, several species in the genus *Campylocentrum* are primarily leafless (e.g., *Campylocentrum pachyrrhizum (Rchb. f.) Rolfe*), as well as all 15 species of *Dendrophylax* [21]. Among these include *D. lindenii* (Lindl.) Bentham ex Rolfe, also known as the ghost orchid. The ghost orchid is restricted to south Florida and western Cuba where it is exceptionally rare [22,23].

As a group, leafless epiphytic orchids have received comparatively little study [24] especially with respect to their ecological interactions with other organisms in situ (e.g., pollinators, mycorrhizal fungi, host tree preferences). Nevertheless, recent studies have linked basidiomycete fungi in the *Ceratobasidiaceae* as primary mycorrhizal associates in several leafless genera throughout Asia and the New World [11,18,25,26,27]. In Florida, the ghost orchid has probably received the most attention exemplified by studies describing the species’ specific habitat and associated flora [22], seed germination [18], pollinators [28,29], floral fragrance and nectar chemistry [30,31], and associated insects [32].

A global review of rare plant reintroductions suggest that outcomes are variable, may take decades to effectively assess, and commonly fail to establish persistent populations. Long-term survival of reintroduced seedlings hinges on interactions with the host tree that involve bark structure and texture, water-holding capacity, surface chemistry, and the ability of roots to physically adhere to the substrate [33]. Root attachment is especially important for leafless orchid species. Likelihood of attachment is also influenced by neighboring epiphytes that may compete for space and light, with subsequent effects on both plant persistence and reproductive output. Among a growing number of studies that involve reintroduction of orchids, resulting establishment varies widely across taxa and geographic regions [34]. Some studies report short-term persistence without recruitment, whereas others demonstrated limited survival beyond initial establishment [34,35]. Experimental introductions of *Dactylorhiza praetermissa* (Druce) Soó, demonstrated that survival and establishment were strongly constrained by ecological factors such as herbivory and vegetation interactions, resulting in poor long-term outcomes [36]. More broadly, global evaluations of orchid translocations indicate that while short-term survival is frequently achieved, natural recruitment and the development of self-sustaining populations remain rare [34]. Imperiled orchid species reintroductions may also be plagued by limited fecundity resulting from the limited genetic diversity that is common in founding populations of rare and threatened plant species [1,34,37]. Orchid reintroduction efforts frequently focus on survival metrics rather than reproductive output or ecological factors, limiting the utility of these trials for long-term conservation planning [5,37].

Here, we performed a longitudinal field survey, spanning seven years, that documented the outcome of a reintroduction effort involving the ghost orchid in Florida. This species has experienced a significant decline during the past two decades due to habitat fragmentation, poaching, hydrological changes, and land development [22,23]. As a flagship species for orchid conservation efforts in the United States, the ghost orchid serves as a useful model for evaluating the challenges and opportunities associated with epiphytic orchid reintroduction efforts, and other leafless orchid species worldwide. We hypothesized that (1) host tree characteristics (species, bark texture) would primarily determine establishment success; (2) root attachment would be necessary for long-term survival; and (3) flowering would occur within 2–3 years and increase over time as plants matured. The present study contributes empirical data to the broader field of plant reintroduction biology, with implications for both ex situ conservation and in situ management of other rare leafless epiphytic orchids.

## 2. Results

### 2.1. Survival

**Figure 1:**
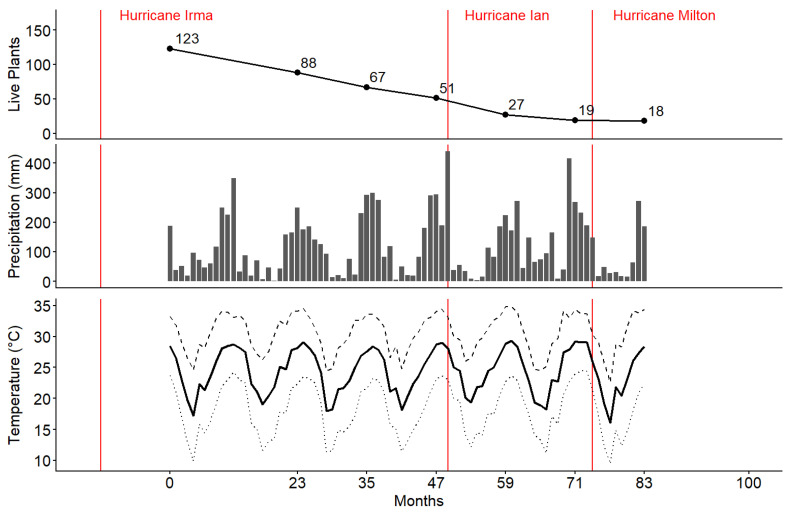
Temporal dynamics of reintroduced *Dendrophylax lindenii* survival and climatic conditions over the 83-month study period. Number of live individuals remaining through time following outplanting in September 2018. Points indicate observed counts at each annual survey interval (**Top**). Monthly precipitation (mm) at the study site (**Middle**). Monthly temperature (°C), including average maximum (dashed line), mean (solid line), and minimum (dotted line) temperatures (**Bottom**). Vertical red lines denote major storm disturbance events during the monitoring period: Hurricane Irma (2017, pre-reintroduction baseline), Hurricane Ian (2022), and Hurricane Milton (2024). PRISM Group, Oregon State University, https://prism.oregonstate.edu, accessed 16 December 2025.

**Figure 2:**
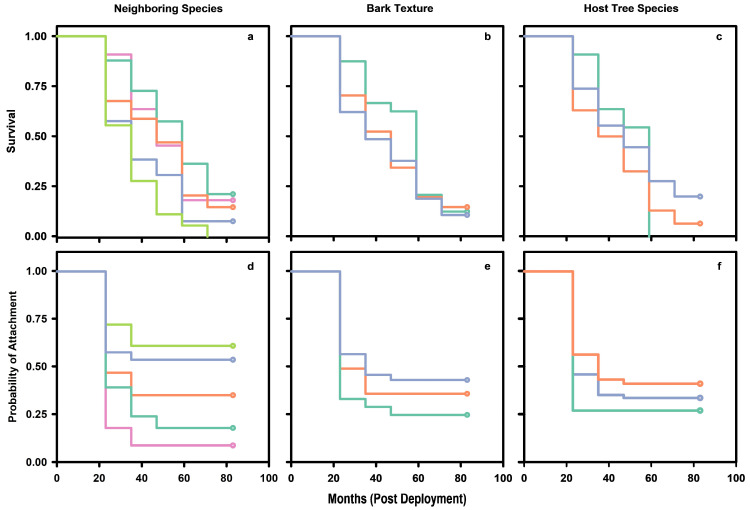
Kaplan–Meier estimates of survival functions for reintroduced ghost orchid post-transplant survival (**a**–**c**) and root attachment (**d**–**f**). Predictors included neighboring species (**a**,**d**), host tree bark texture (**b**,**e**), and host tree species (**c**,**f**). Neighboring species included other ghost orchids (pink), moss (cyan), lichen (orange), bromeliads (blue), and ferns (green). Bark textures included smooth (cyan), semi-corrugated (orange), and corrugated (blue). Host tree species included bald cypress (cyan), pop ash (orange), and pond apple (blue). Ninety-five percent point-wise confidence intervals omitted for clarity.

Of the 123 Ghost Orchid plants reintroduced in 2018, 18 remained alive in 2025 after 83 months, reflecting an 85.4% reduction in live individuals over time despite periodic disturbance (Figure 1). Survival probability ranged from 0 to 67% over 83 months. Median survival time ranged from 23 to 71 months with an average of 44 months, 95% CI [43.2, 45.3] (Figure 2a–c, Table A1). The log rank test provided sufficient statistical evidence to reject the null hypothesis that survival curves were the same when stratifying by neighbor type, bark texture, and host tree species ($\chi_{28}^{2}$ = 50.96, *p* = 0.005). Cox regression indicated that neighbor type ($\chi_{4}^{2}$ = 16.88, *p* = 0.0020) was a statistically significant predictor of reintroduced ghost orchid survival. However, host tree species was on the cusp of statistical significance ($\chi_{2}^{2}$ = 5.81, *p* = 0.0546), while bark texture ($\chi_{2}^{2}$ = 0.90, *p* = 0.6370) was not a statistically significant predictor variable.

The presence of ferns had a strong effect on the survival of outplanted ghost orchids. For example, the mortality risk was 2.2–3.8 times greater when a ghost orchid was outplanted near a fern compared to another ghost orchid, moss, or lichen respectively. Similarly, the likelihood of dying was two times greater when a ghost orchid was planted adjacent to a moss rather than a bromeliad (Table 1). Comparisons between host trees reveal that the likelihood of death was 1.6 times greater when a ghost orchid was outplanted on a pop ash compared to pond apple. Otherwise, there was no statistically significant effect of host tree species on survival (Table 1).

### 2.2. Attachment

Root attachment occurred within the first two years, with 93% of surviving plants having at least one root attached. Despite overall population decline, attachment remained high. For example, in 2025, 17 of 18 surviving plants (94%) had attached roots. Over 90% of surviving plants displayed root attachment from 35 months onward. It was observed that for surviving individuals, temporal trends in root length, number and proportion of aerial vs. attached roots were not constant but rather fluctuated over the study period.

The probability of no root attachment ranged from 0 to 100% over 83 months. Median time to root attachment ranged from 23 to 35 months with an average of 26.2 months, 95% CI [25.7, 26.6] (Figure 2d–f, Table A2). The log rank test provided sufficient statistical evidence to reject the null hypothesis that temporal patterns of root attachment were the same when stratifying by neighbor type, bark texture, and host tree species ($\chi_{28}^{2}$ = 48.3957, *p* = 0.0097). Cox regression indicated that neighbor type ($\chi_{4}^{2}$ = 13.2971, *p* = 0.0099), but not host tree species ($\chi_{2}^{2}$ = 0.1279, *p* = 0.9381), or bark texture ($\chi_{2}^{2}$ = 0.2146, *p* = 0.8983) were statistically significant predictors of ghost orchid root attachment (Table 1). Neighboring plants like other ghost orchids, mosses, and lichens exerted the largest effects on reintroduced ghost orchid root attachment compared to bromeliads and ferns. For instance, the likelihood of root attachment was about 3.5 to 4.6 times higher when a reintroduced ghost orchid was transplanted next to another ghost orchid compared to a bromeliad or fern. Similarly, the likelihood of root attachment was about 2.0 to 3.0 times greater when a ghost orchid was transplanted next to a moss or lichen compared to bromeliad or fern. Otherwise, the remaining comparisons were not statistically significant (Table 1).

### 2.3. Reproduction

**Figure 3:**
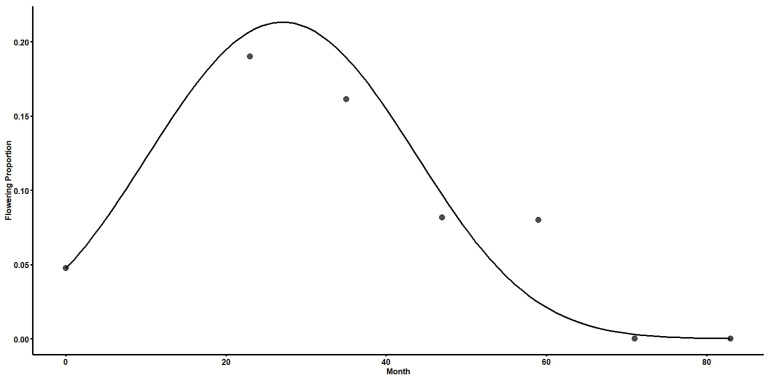
Temporal flowering probability of reintroduced *Dendrophylax lindenii* over 83 months following outplanting. Points represent the observed proportion of surviving individuals flowering at each survey interval. The solid curve represents the fitted generalized linear mixed-effects model (binomial distribution with logit link) including linear and quadratic components of scaled month.

The proportion of flowering increased from 0.05–0.19 between the initial reintroduction and 23 months post-transplanting. Flowering proportion decreased at subsequent observation periods until no flowers were observed in months 71 and 83 (Figure 3). Our model confirmed that the quadratic model (AIC = 212.97, Table A3) in proportion of flowering was the best fitting model when compared to linear (AIC = 234.54) or cubic models (AIC = 214.92). The overall flowering rate during the study period was 10.1% and peak flowering was estimated to occur at 28.9 months (Figure 3).

Analysis of marginal means indicated that the probability of flowering was highest when a ghost orchid was transplanted next to other ghost orchids (25.4%). The probability of flowering did not surpass 6% when other adjacent plant species were present. Similarly, the probability of flowering remained lower when considering host tree or bark effects (range 3.7–13.3%). Our model detected statistically significant neighboring species effect but not host tree or bark effects (Table 2). For example, the odds of flowering for reintroduced ghost orchids when planted next to lichen were 11% compared to the odds of flowering when planted next to other ghost orchids. No other comparisons were statistically significant. However, the relatively large host tree and bark effects should be interpreted with caution given the wide confidence intervals.

## 3. Discussion

Rare epiphytic orchid reintroduction is an increasingly important conservation strategy as natural populations continue to decline due to habitat fragmentation, hydrologic alteration, climate change, and poaching. However, reintroduction success remains difficult to predict because epiphytes depend on a complex suite of microhabitat and biotic interactions that are rarely evaluated over long time frames [34,35,37]. To address this gap, we conducted a seven-year field study to evaluate long term survival, root attachment, and reproduction of 123 reintroduced leafless epiphytic ghost orchids. Such extended monitoring datasets remain rare for orchid reintroductions, which are typically assessed over short post-planting periods, despite the slow demographic responses characteristic of orchids [5,34]. We found that although overall survival declined substantially through time, most surviving individuals successfully attached to host tree bark within the first two years and remained attached thereafter. A central and unanticipated result of this study was that neighbor identity emerged as the strongest predictor of both survival and root attachment, whereas host tree species and bark texture played comparatively minor roles. Flowering was infrequent and declined through time, indicating that successful reproduction remains a major bottleneck for population persistence. Although several capsules were observed during the study, none developed to full maturity, indicating that the absence of fruit set further constrains reproductive output and long-term population replacement. Together, these results demonstrate that fine-scale neighbor interactions dominate establishment outcomes for leafless epiphytic orchids, and that reproductive failure due to genetic or pollinator constraints represents an insurmountable barrier to self-sustaining reintroduced populations under current management approaches.

### 3.1. Influence of Neighboring Epiphytes on Survival

**Figure 4:**
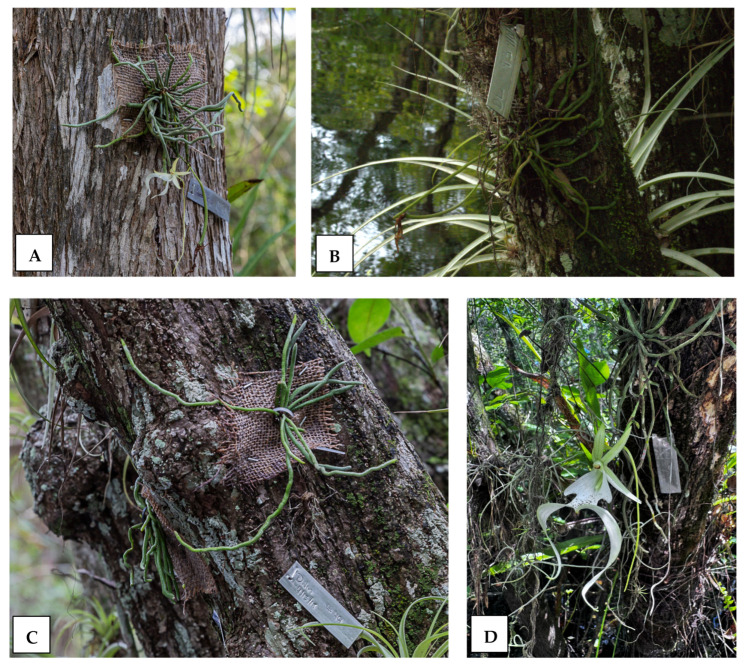
Reintroduction of the ghost orchid on the Florida Panther National Wildlife Refuge (2018). Orchids were grown onto burlap and affixed to trees with zip ties and staples (**A**). Plants were observed to flower and fruit throughout the seven-year study period (**A**,**B**,**D**). Neighboring species such as moss (**B**), lichen (**A**), and bromeliads (**B**,**C**) are visible near reintroduced plants. Root attachment and green growing root tips visible in (**B**,**C**). Photo credit L. Richardson (**A**) A. Herdman (**B**–**D**).

Neighboring vegetation exerted strong effects on transplanted ghost orchid survival. Ghost orchids situated near ferns experienced higher mortality compared with those near mosses, lichens, or other ghost orchids. These observed neighbor effects may reflect underlying microclimate gradients rather than direct biotic interactions, i.e., ferns might occupy darker microsites inhospitable to ghost orchid persistence. Alternatively, neighbor effects may represent direct biotic interactions. For instance, survival was markedly higher when plants were placed adjacent to bryophytes (Figure 4). These patterns are consistent with the well-documented facilitative role of bryophytes in epiphyte establishment. For example, moss mats function as “safe sites” by retaining moisture, buffering temperature fluctuations, trapping organic particulates, and stabilizing seed and root placement [38,39,40]. Experimental transplant studies in tropical forests have shown that orchid and other vascular epiphyte seedlings exhibit higher survival and attachment when placed in bryophyte-covered microsites than on bare bark, largely due to improved water availability during dry periods [37,39,40,41].

Lichens appear to confer intermediate benefits by providing surface roughness and some microclimatic buffering. Lichens may lack the moisture-retention capacity and organic matter accumulation characteristic of mosses. This may limit the ability of lichens to function as long-term nurseries for vascular epiphytes [38,42]. By contrast, ferns and large bromeliads frequently suppress establishment of smaller epiphytes by monopolizing space and intercepting light and rainfall before it reaches the bark surface [42,43,44]. Large bromeliads, in particular, have been shown to restructure branch-level hydrology and nutrient interception in ways that disadvantage small orchids and bryophyte-associated species [44]. Epiphytic ferns can form dense root mats that physically limit orchid seedling attachment sites. Together, these studies support the interpretation that bryophyte dominated microsites promote ghost orchid survival through facilitation, whereas fern and bromeliad dominated microsites impose competitive or physiologically stressful conditions. Our findings indicate that the identity of neighboring epiphytes can act as a reliable indicator of microhabitat quality, with moss and lichen dominated patches offering particularly favorable conditions for ghost orchid establishment.

### 3.2. Mechanisms Underlying Moss and Lichen-Driven Facilitation

The enhanced survival near mosses and lichens likely arises from both direct and indirect mechanisms. Directly, mosses buffer desiccation, maintain more stable humidity and temperature regimes, and trap nutrient-rich debris, thereby creating conditions conducive to the growth of leafless epiphytes reliant on exposed root systems [38]. Lichens provide some structural stability and incident light moderation, though they lack mosses’ water-holding capacity. Beyond these physical mechanisms, bryophytes may also impart a nurse-plant-like function [45]. Nurse plants typically reduce abiotic stress by moderating temperature extremes, increasing water availability, stabilizing substrates, and enhancing nutrient concentrations within microsites, thereby increasing recruitment success in otherwise harsh environments [45,46]. In this context, moss mats facilitate the establishment of other plants by improving local microclimates and modifying moisture or nutrient conditions, closely paralleling patterns observed in this study.

However, these neighbor effects may also reflect underlying microclimatic gradients. Ferns, for instance, often occupy darker, more humid microsites, and it remains possible that the poor survival of ghost orchids near ferns reflects low-light conditions or direct biotic suppression. Future studies that integrate fine-scale measurements of light, humidity, and bark moisture would help disentangle the degree to which neighbor identity versus microclimate drives the observed differences.

### 3.3. Mycorrhizal Associates: A Way Forward?

Previous studies that cover mycorrhizal associates [11,18] determined that the ghost orchid has a high specificity for a single *Ceratobasidium* species (OTU). Whether or not this fungus is associated with non-orchids, such as mosses, growing in close proximity to the ghost orchid, remains unknown. This prospect deserves further inquiry considering that bryophytes and lichen mats host distinct and diverse fungal assemblages, including known orchid mycorrhizal taxa [47,48,49]. Moss-rich microsites may serve as reservoirs of compatible mycorrhizae or may create microclimates that allow fungi to persist longer after rainfall pulses. Using molecular techniques (amplicon sequencing), a previous study [11] sampled the roots of several outplanted ghost orchid seedlings from 2015 and determined that the *Ceratobasidium* species associated with this orchid were largely absent. This finding suggests that this fungus may be acquired earlier in the seed germination process, and/or that established roots may require considerable time (i.e., >three years) to recruit *Ceratobasidium* fungi from the surrounding substrate community.

### 3.4. Root Attachment as a Weak Predictor of Persistence

Although root attachment was widespread, exceeding 90% by month 35, it was not a significant predictor of long-term ghost orchid survival (Figure 4). This pattern suggests that once a minimum threshold of mechanical anchorage is achieved, other microenvironmental factors such as mycorrhizal access and moisture dynamics become primary determinants of persistence. Despite this, put plainly, if the orchid is not able to utilize the physical structure of the host tree, survival is not possible. Thus, all interpretations should take this into account because host trees provide more than just a substrate for epiphytes (i.e., canopy). At the microsite level root attachment was nonetheless more common near mosses and lichens, possibly due to their superior water-holding capacity and the humid boundary layer they maintain at the bark surface [50,51]. This concept is supported by a previous study [22] who noted that more than half of all ghost orchids in both Florida and Cuba originated on bark surfaces with a northern orientation where direct sun exposure and presumably water loss rates would be more limited. Bryophyte mats have repeatedly been shown to stabilize moisture availability for epiphytic roots during dry periods and to prolong hydration following rainfall, thereby promoting sustained root growth and adhesion [40,42]. Lichens may facilitate attachment through increased surface roughness and microtopographic complexity, but their limited moisture retention likely constrains their facilitative role relative to mosses. We acknowledge that this explanation remains partly speculative for ghost orchids, as bark chemistry and microtopography were not directly quantified in this study. However, the strong dominance of neighbor identity over host tree effects suggests that local biotic and hydrologic conditions at the bark surface override broader host-tree-level controls in shaping attachment success and early persistence.

In contrast, dense concentrations of ferns and bromeliads may reduce opportunities for root attachment by intercepting rainfall before it reaches the bark surface, altering stemflow patterns, and reducing bark-level moisture availability [42,44]. Large bromeliads are known to restructure local hydrology by capturing canopy water runoff within their spirally arranged and basally attached leaves. This leaf arrangement forms water holding tanks that effectively divert moisture away from adjacent bark microsites that would otherwise support orchid root growth. Epiphytic ferns can further suppress attachment by forming dense root mats that physically exclude orchid roots from direct bark contact and alter microbial communities at the bark interface [42,43]. Together, these mechanisms provide strong physiological and physical explanations for the reduced attachment observed near fern- and bromeliad-dominated microsites in this study.

Overall, host tree species and bark texture did not statistically influence attachment or survival, possibly because hydrology and slough structure exert stronger environmental controls at this site than bark-level traits. This interpretation is consistent with the broader epiphyte literature demonstrating that bark roughness and chemistry interact strongly with moisture regimes, fungal distributions, and canopy hydrodynamics, causing bark effects to be highly context dependent [33]. Studies of non-orchid epiphytes further show that host specificity often weakens in hydrologically stable or perennially humid systems, where persistent moisture reduces the selective importance of bark traits [43]. However, the strong dominance of neighbor identity over host tree effects suggests that local biotic and hydrologic conditions at the bark surface override broader host-tree-level controls in shaping attachment success and early persistence.

### 3.5. Reproductive Constraints and Long-Term Viability

Flowering was infrequent and declined steadily over time, with peak flowering proportion (19%) occurring early in the experiment. Over the duration of the monitoring, 36 flowers in total were recorded, peaking in month 23 at 15 flowers. This pattern mirrors observations in wild ghost orchid populations, where flowering and reproductive output often track hydroperiod, storm disturbance, and short-term environmental differences in hydroperiod [22,52]. Early flowering may also reflect stress responses or residual carryover effects from greenhouse acclimatization, which have been documented in reintroduced orchids and other epiphytes [5,34]. Most notably, none of the immature capsules continued to develop, which is consistent with prior greenhouse observations of capsule formation, but no mature seed was produced in ghost orchids [50]. Field monitoring did not include development of the seed capsules over time to determine when development aborted and may be an object for future studies that face this constraint.

Several mechanisms may contribute to this reproductive bottleneck: (1) Inbreeding depression represents a likely explanation, as all reintroduced individuals originated from a single self-pollinated capsule. This genetic bottleneck is a known risk factor that reduces seed viability and reproductive success in rare orchids [35,37]; (2) insufficient or inconsistent pollinator visitation, which is recognized as a major constraint for highly specialized epiphytic orchids [5,22]; and (3) resource or fungal limitations, as fruit maturation in orchids is tightly linked to carbon and nutrient supply mediated by orchid mycorrhizal fungi [11,18]. Regardless of mechanism, the absence of successful fruit maturation and concomitant seed production strongly suggests that this reintroduced population is unlikely to become self-sustaining without additional genetic or ecological intervention [35,53].

### 3.6. Conservation Implications and Recommendations

Collectively, these findings underscore several key considerations for future ghost orchid restoration and leafless epiphyte conservation efforts more broadly. First, microsite selection is critical: prioritizing moss or lichen dominated bark patches while avoiding fern or bromeliad zones may substantially improve survival and attachment outcomes. This microsite screening criterion is a simple yet powerful addition to reintroduction planning. Second, fungal assessments should be integrated into site selection and post-planting monitoring, building on evidence that orchid mycorrhizal fungi (OMF) availability strongly shapes establishment success [11,18]. Third, greater attention to genetic diversity is needed, as reliance on a single maternal line may have constrained reproductive potential. In particular, providence and diversity of material will become increasingly more important due to the proliferation of tissue culture as a tool in conservation. Finally, these results highlight broader lessons for epiphytic orchid reintroduction: neighbor effects can mediate outcomes as strongly as host tree characteristics, adding an important dimension to restoration planning that has been largely overlooked in past efforts [5,34].

Despite the mechanistic insights gained from this study, the demographic outcomes warrant caution in the application of reintroduction as a primary conservation strategy for *Dendrophylax lindenii*. The combination of high long-term mortality and the absence of successful reproduction indicates that reintroduction, as presently implemented, is unlikely to produce self-sustaining populations. Future efforts should incorporate explicit microsite screening based on neighboring epiphyte composition, prioritize genetic diversification of propagated cohorts, and integrate fungal availability assessments into both site selection and post-deployment monitoring. Without these modifications, additional reintroductions will likely replicate patterns of persistence without recruitment observed here. Moving forward, prioritizing conservation actions that protect existing wild individuals, further assessment of existing population genetics, and pursuing further reintroduction apart from wild sites remain the most reliable strategies for near-term species persistence.

### 3.7. Conclusions

This long-term reintroduction trial demonstrates that microsite-level biotic interactions shape the persistence of the leafless epiphytic species *Dendrophylax lindenii*. Bryophyte- and lichen-dominated microsites consistently promoted survival and attachment, whereas proximity to ferns and bromeliads inhibited establishment. Root attachment, although rapid and widespread, was not sufficient to ensure long term survival, and low reproductive output remains a major barrier to population viability. These results reinforce the idea that host tree bark presents a mosaic of epiphytic patches shaped by local competitive and facilitative interactions [54]. Environmental filtering therefore occurs not only at the level of the host tree species, but also at very fine spatial scales, where bark microtopography and nearby epiphytes influence local resource availability [33]. These findings underscore the necessity of extended monitoring and illustrate the value of integrating microhabitat ecology, fungal symbioses, and genetic considerations into future orchid restoration programs. Our study highlights the importance of in-field investigation on native flora for conservation purposes [55]. Ultimately, this case study contributes to a more nuanced understanding of epiphytic orchid reintroduction and highlights both the potential and challenges of restoring species with highly specialized ecological requirements. Further, as reintroduced cohorts may not develop into persistent, self-sustaining populations, these findings further elevate the importance of protecting and conserving remaining naturally occurring ghost orchid populations to this species’ survival.

## 4. Materials and Methods

### 4.1. Study Site and Species

**Figure 5:**
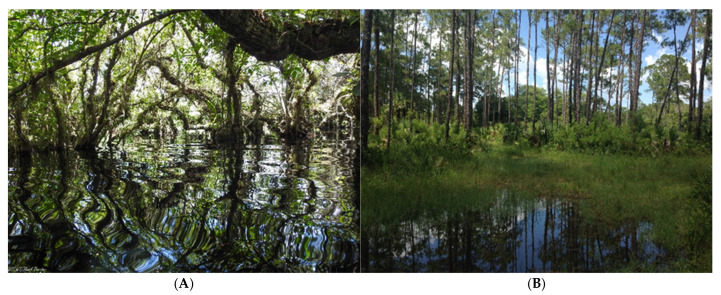
The ghost orchid resides in (**A**) seasonally flooded swamps and slough in southwestern Florida. The habitat changes sharply to (**B**) mesic pinewoods and cypress domes with the change in hydrology. This habitat distribution restricts site colonization and makes populations sensitive to habitat fragmentation, poaching, and climate change.

Native epiphytic orchid communities in the United States are restricted to the state of Florida, where a unique confluence of subtropical climate and hydrology support a diverse orchid flora. Florida’s southern peninsula, characterized by a Köppen–Geiger climate classification of Am/Aw (tropical monsoon and tropical savanna), provides a refuge from subfreezing temperatures and fire regimes that otherwise restrict epiphytic distributions in temperate regions. This combination of climate and hydrology supports the pond apple slough ecosystem, a rare and hydrologically sensitive wetland community considered globally and regionally vulnerable to disturbance and decline [56]. A Pond-Apple/Pop Ash slough is a unique wetland system found in south Florida, and is characterized by long hydroperiods, deep muck soils, and overstory vegetation dominated by Pop Ash (*Fraxinus caroliniana* Mill. (Oleaceae), Bald Cypress (*Taxodium distichum* (L.) Rich. (Cupressaceae), and Pond Apple (*Annona glabra* L. (Annonaceae). They are often embedded within other palustrine wetlands (e.g., mixed hardwood strand forest), and on the Florida Panther National Wildlife Refuge (FPNWR), they occupy the lowest elevations and contain the deepest muck soils. Epiphyte communities and diversity increase in the deepest portions of swamps and sloughs [16,56]. These communities are bounded by mesic pine flatwoods dominated by Slash Pine (*Pinus elliottii* Engelm), Saw Palmetto (*Serenoa repens* (W. Bartram) Small), and Cabbage Palm (Sabal palmetto (Walt.) Lodd. ex J.A. & J.H. Schultes); ecosystems characterized by frequent fire intrusion. The sharp contrast in fire and hydrology between adjacent habitats limits epiphytic orchids to the protected central sloughs (Figure 5). Water levels in pond apple sloughs fluctuate seasonally. Water depth in the summer can be up to ~1 m, while water may completely recede during the winter leaving dry soils [22,57].

Population viability analyses (PVAs) confirm that ghost orchid populations within the FPNWR, which is adjacent to the Fakahatchee Strand, are below replacement levels, suggesting long-term decline without intervention [57]. FPNWR was selected as a site for reintroduction for this reason, but also because access remains highly controlled as it remains closed to the public, and the location falls within the current range of the ghost orchid. Declines have also been observed in parallel monitoring efforts of ghost orchids in Cuba and for the ribbon orchid (*Campylocentrum pachyrrhizum* (Rchb. f.) Rolfe) in Florida [52,58], indicating broader patterns of vulnerability among native epiphytic orchids. These declines are associated with persistent and interacting stressors, including poaching, increased tropical storm activity, saltwater intrusion, altered fire regimes, and drought.

### 4.2. Plant Propagation and Plant Material

We introduced 123 cultivated ghost orchids into a natural pond apple slough during September 2018, using attachment techniques developed along with earlier experimental plantings in 2015 and 2016 (M. E. Kane, unpublished data), to further evaluate the efficacy of ghost orchid reintroduction. Ghost orchid seeds were collected opportunistically from one mature wild individual located within the Florida Panther National Wildlife Refuge (26.1925° N, 81.4051° W) in Collier County, FL. Seedlings were produced through asymbiotic in vitro germination [18]. Collecting seeds from wild populations is a common strategy for preserving rare species [59,60]. While we acknowledge that clonal or sibling relatedness of these individuals may constrain reproductive compatibility and limit extrapolation of reproductive metrics to genetically diverse populations, the uncommon availability of these plants presented a timely and critical opportunity to test possible reintroduction outcomes for this imperiled species. Plants were transferred to greenhouse conditions following 3.5 years of growth in vitro. Seedlings were acclimatized under shade cloth (50% light transmittance) and maintained with frequent misting to simulate high-humidity microhabitats typical of slough environments [50,61]. Each plant was affixed to a temporary matrix in the form of a tight weave burlap sheet in the greenhouse prior to outplanting. The burlap material was chosen because it holds moisture and decomposes gradually, supporting root attachment while minimizing physical or chemical disturbance to the plant or the host substrate.

### 4.3. Planting and Initial Attachment

Plants selected for reintroduction had well-developed root systems that had grown into or through the temporary matrix burlap sheet during the growth period in the greenhouse. Burlap sheets were trimmed to 12 cm × 12 cm around the root mass. Each reintroduced orchid was affixed to the trunk or major branches of the host tree by securing the burlap sheet to the tree bark using small steel staples (Figure 4). Orchids were affixed at heights ranging from 1 to 2 m above the ground level in shaded to semi-shaded conditions typical of extant ghost orchid populations.

### 4.4. Survey Methods

The reintroduced population was surveyed annually in July for seven years, when the species was most reproductively active (i.e., initiated anthesis). Each orchid was tagged and geolocated at the time of planting to ensure consistent monitoring. Annual surveys followed species-specific protocols established by PVA on the FPNWR [52,57,58]. Survival was assessed by classifying individuals as dead when persistent necrotic tissue was observed at the central meristematic region where new growth is initiated. To reduce the likelihood of misclassification, individuals recorded as dead were revisited in subsequent survey years. No plants classified as dead exhibited recovery or renewed growth.

Root attachment was documented as the presence of at least one root physically adhered to the host substrate. Burlap was observed to decompose over time and did not impact and did not impair data collection. Reproductive status was monitored annually by recording the presence of flowers or developing seed capsules. Neighboring epiphytic species within 5 cm of each outplanted orchid were noted. This distance was selected to standardize microsite characterization, as complete isolation from neighboring epiphytes is not feasible on natural bark substrates. Neighboring taxa were recorded as presence/absence rather than quantified by cover or abundance.

We recorded the host tree species along with the bark texture at the site of attachment. Host trees include *Annona glabra*, *Taxodium distichum*, and *Fraxinus caroliniana.* Bark texture was classified as smooth, semi-corrugated, and corrugated (Figure 4).

### 4.5. Data Analysis

#### 4.5.1. Survival and Root Attachment

Non- and semi-parametric time-to-event analyses were utilized to assess two ghost orchid response variables, survival and root attachment. Predictor variables included host tree species, type of neighboring vegetation, and bark texture. Ghost orchid death and root attachment events during the study interval were coded as 1 and censored events as 0. Censored events included ghost orchids that survived or did not have roots that attached during the study period. Kaplan–Meier estimates of survivor functions were stratified by host tree species, neighbor type, and bark texture. We used Kaplan–Meier estimates to generate temporal survival curves and calculate median death and root attachment time (Figure 2). The log-rank statistic was used to test the null hypothesis of no difference in temporal patterns when stratifying by host trees, neighboring vegetation, and bark type.

The effects of predictors on the likelihood of ghost orchid survival and root attachment were modelled using Cox regression and the exact method to account for tied event times. The proportional hazards assumption was evaluated graphically and with residual analysis prior to model building. These analyses did not detect violations of the proportional hazard assumption. We constructed orthogonal linear contrasts to test the null hypothesis that coefficients for treatment comparisons were equal and estimated the hazard of death or root attachment with hazard ratios (Table 1). Time-to-event analyses were conducted using SAS Version 9.4 (SAS Institute Inc., Cary, NC, USA, 2023).

#### 4.5.2. Reproduction

We evaluated the proportion of plants flowering over the 83-month survey period. Capsules were observed in 2018, 2021 and 2024. However, none of the capsules developed to maturity. Therefore, all subsequent analyses of reproduction are limited to flower production as a response variable. Preliminary analyses revealed that reintroduced ghost orchids associated with ferns and other orchids did not produce flowers during the study period. Consequently, we removed these variables from the analysis. Moreover, variance inflation factor and tolerance values ranged from 1.03–2.12 and 0.47–0.97 respectively, indicating a lack of multicollinearity for our time and predictor variables (Table 2).

Subsequently, we tested linear, quadratic, and cubic time trends of flowering utilizing a generalized linear mixed model with a binomial distribution, and logit link function with the glmer package in Rstudio (version 4.5). We scaled the month variable by subtracting the mean month from each time point then dividing by the standard deviation with the scale function. We held scaled month, neighbor, and bark as fixed effects and individual plants as the random effect. Maximum likelihood estimation utilized the Laplace approximation and optimization was through bound optimization by quadratic approximation (bobyqa). We compared linear, quadratic, and cubic models with Akaike Information Criterion (AIC). We used the DHARMa package to conduct model diagnostics and test for temporal autocorrelation of residuals. The tests did not detect issues with distribution (KS test *p* = 0.23), dispersion (dispersion test *p* = 0.42), outliers (outlier test *p* = 1.00), or temporal autocorrelation (Durbin–Watson test *p* = 0.27) (Figure 3). We generated marginal means with the emmeans package and calculated odds ratios with 95% CI manually.

## Figures and Tables

**Table 1:** Orthogonal contrasts from Cox proportional hazards models evaluating survival (mortality risk) and root attachment of reintroduced *Dendrophylax lindenii* across neighboring epiphyte communities and host tree species. The hazard ratio (HR) represents the relative risk of the event (death in the survival dataset; root attachment in the attachment dataset) for the first group listed relative to the second group. The 95% confidence interval (95% CI) provides the precision of the HR estimate; intervals that do not include 1 indicate statistical significance at α = 0.05. Reciprocal hazard ratios (1/HR) and reciprocal confidence intervals (1/CI) are provided to facilitate ecological interpretation when HR < 1, allowing direct expression of how many times greater the hazard is in the comparison group. Survival dataset HR values reflect relative mortality risk. Attachment dataset HR values reflect relative probability of root attachment through time.

Contrast	HR	95 CI	1/HR	1/CI
***Survival dataset***				
*Neighbor comparisons*				
ghost orchid v. moss	1.414	0.614, 3.253	0.71	
ghost orchid v. lichen	0.811	0.364, 1.808	1.23	0.55, 2.75
ghost orchid v. bromeliad	0.692	0.307, 1.561	1.45	0.64, 3.26
**ghost orchid v. fern**	**0.369**	**0.154, 0.882**	**2.71**	1.13, 6.49
moss v. lichen	0.574	0.328, 1.005	1.74	0.99, 3.05
**moss v. bromeliad**	**0.490**	**0.271, 0.883**	**2.04**	1.20, 3.69
**moss v. fern**	**0.261**	**0.135, 0.506**	**3.83**	1.98, 7.41
lichen v. bromeliad	0.853	0.478, 1.522	1.17	0.66, 2.09
**lichen v. fern**	**0.455**	**0.242, 0.854**	**2.19**	1.17, 4.13
bromeliad v. fern	0.533	0.275, 1.034	1.87	0.97, 3.64
*Host tree comparisons*				
cypress v. pop ash	1.289	0.487, 3.415	0.78	
cypress v. pond apple	2.104	0.809, 5.474	0.47	
**pop ash v. pond apple**	**1.632**	**1.040, 2.561**	**0.6127**	
*****Attachment dataset*****				
*Neighbor comparison*				
ghost orchid v. moss	1.580	0.669, 3.731	0.63	
ghost orchid v. lichen	2.305	0.995, 5.340	0.43	
**ghost orchid v. bromeliad**	**3.521**	**1.3720, 9.039**	0.28	
**ghost orchid v. fern**	**4.622**	**1.620, 13.192**	0.21	
moss v. lichen	1.459	0.796, 2.674	0.69	
**moss v. bromeliad**	**2.229**	**1.097, 4.528**	0.45	
**moss v. fern**	**2.926**	**1.221, 7.014**	0.34	
lichen v. bromeliad	1.528	0.736, 3.171	0.65	
**lichen v. fern**	**2.005**	**0.825, 4.873**	0.50	
bromeliad v. fern	1.313	0.509, 3.388	0.76	

^z^ Bold indicates comparisons that were found to be significant.

**Table 2:** Model-estimated flowering probabilities of reintroduced *Dendrophylax lindenii* across neighboring epiphyte communities, host tree species, and bark texture categories. Probabilities represent marginal means derived from the generalized linear mixed-effects model (binomial distribution with logit link), averaged across other predictors and time. SE denotes the standard error of the estimated probability. CI represents the 95% confidence interval for the predicted probability on the response scale. Higher values indicate greater predicted likelihood of flowering during the study period.

Predictor	Probability	SE	CI
*Neighbor*			
ghost orchid	0.2538	0.1440	0.602
moss	0.0613	0.0432	0.221
lichen	0.0375	0.0297	0.163
bromeliad	0.0613	0.0297	0.244
*Host Tree*			
cypress	0.0369	0.0419	0.279
pop ash	0.1112	0.0656	0.315
pond apple	0.1198	0.0627	0.304
*Bark Texture*			
smooth	0.1334	0.0814	0.380
semi-corrugated	0.0759	0.0490	0.244
corrugated	0.0490	0.0383	0.205

## Data Availability

The data presented in this study are available on request from the corresponding author. The data are not publicly available due to the rarity of the species investigated and to limit the access to location data.

## Appendix A

**Table A1:** Kaplan–Meier survival metrics for reintroduced *Dendrophylax lindenii*, stratified by neighboring epiphyte community, host tree species, and bark texture. Survival % represents the proportion of individuals remaining alive at the final observation (83 months). *n* denotes the number of individuals within each stratum. T25, T50, and T75 indicate the estimated time (months post-transplant) at which 25%, 50%, and 75% mortality occurred, respectively, with 95% confidence intervals (CI) shown in parentheses. Survival rate is expressed as the reciprocal of the median survival time (1/T50), providing a standardized measure of mortality intensity that facilitates comparison among strata; higher values indicate faster mortality. Dashes indicate that percentile estimates could not be calculated due to right-censoring or insufficient events.

					Percentile Estimates (95% CI)			
Neighbor	Bark Texture	Host Tree	Survival %	*n*	*T*_25_	*T*_50_	*T*_75_	Rate (1/*t*_50_*)*
ghost orchid	smooth	cypress	0	4	41(35, -)	53 (35, - ^z^)	59 (35, -)	0.02
ghost orchid	semi-corrugated	pond apple	0	1	35 (-, -)	35 (-, -)	35 (-, -)	0.03
ghost orchid	corrugated	pop ash	50	4	41 (23, -)	- ^y^	-	0.02
ghost orchid	corrugated	pond apple	0	2	35 (35, -)	41 (35, -)	47 (35, -)	0.02
moss	smooth	cypress	0	2	35 (35, -)	47 (35, -)	59 (35, -)	0.02
moss	semi-corrugated	cypress	0	2	59 (59, -)	65 (59, -)	71 (59, -)	0.02
moss	semi-corrugated	pop ash	37.5	8	35 (23, 47)	47 (23, -)	-	0.02
moss	semi-corrugated	pond apple	16.67	6	59 (23, 59)	59 (23, -)	71 (59, -)	0.02
moss	corrugated	cypress	25	4	29 (23, 71)	53 (23, -)	-	0.02
moss	corrugated	pop ash	25	8	41 (23, 59)	53 (23, -)	-	0.02
moss	corrugated	pond apple	33.33	3	47 (47, -)	71 (47, -)	-	0.01
lichen	smooth	cypress	0	3	23 (23, -)	35 (23, -)	59 (23, -)	0.03
lichen	semi-corrugated	cypress	0	2	59 (-, -)	59 (-, -)	59 (-, -)	0.02
lichen	semi-corrugated	pop ash	0	3	23 (23, -)	23 (23, -)	47 (23, -)	0.04
lichen	semi-corrugated	pond apple	0	4	35 (23, 47)	47 (23, -)	59 (23, -)	0.02
lichen	corrugated	cypress	66.67	3	59 (59, -)	-	-	
lichen	corrugated	pop ash	18.75	16	23 (23, 35)	35 (23, 59)	65 (35, -)	0.03
lichen	corrugated	pond apple	0	3	59 (-, -)	59 (-, -)	59 (-, -)	0.02
bromeliad	smooth	cypress	0	2	59 (-, -)	59 (-, -)	59 (-, -)	0.02
bromeliad	semi-corrugated	cypress	0	1	23 (-, -)	23 (-, -)	23 (-, -)	0.04
bromeliad	semi-corrugated	pop ash	0	5	35 (23, 47)	47 (23, -)	47 (23, -)	0.02
bromeliad	semi-corrugated	pond apple	16.75	6	23 (-, -)	23 (23, -)	35 (23, -)	0.04
bromeliad	corrugated	pop ash	0	9	23 (23, 35)	35 (23, -)	35 (23, -)	0.03
bromeliad	corrugated	pond apple	66.67	3	23 (23, -)	-	-	
fern	semi-corrugated	pop ash	0	2	35 (35, -)	41 (35, -)	47 (35, -)	0.02
fern	semi-corrugated	pond apple	0	6	23 (-, -)	23 (-, -)	23 (-, -)	0.04
fern	corrugated	cypress	0	1	35 (-, -)	35 (-, -)	35 (-, -)	0.03
fern	corrugated	pop ash	0	6	35 (23, 47)	47 (23, -)	59 (35, -)	0.02
fern	corrugated	pond apple	0	3	23 (23, -)	35 (23, -)	35 (23, -)	0.03
		Average (95% CI)	12.3 (10.9, 13.7)			44.2 (43.2, 45.3)		0.02 (0.02, 0.03)

^z^ Upper confidence interval does not vary from lower confidence interval. ^y^ The Kaplan–Meier estimator did not reach a failure probability >0.50 or 0.75.

## Appendix B

**Table A2:** Kaplan–Meier root attachment metrics for reintroduced *Dendrophylax lindenii*, stratified by neighboring epiphyte community, host tree species, and bark texture. Attach % represents the proportion of individuals achieving root attachment by the final observation (83 months). *n* denotes the number of individuals within each stratum. T_25_, T_50_, and T_75_ indicate the estimated time (months post-transplant) at which 25%, 50%, and 75% of individuals achieved root attachment, respectively, with 95% confidence intervals shown in parentheses where estimable. Attachment rate is expressed as the reciprocal of the median attachment time (1/T_50_), providing a standardized measure of attachment speed that facilitates comparison among strata; higher values indicate more rapid attachment. Dashes indicate that percentile estimates could not be calculated due to right-censoring, insufficient events, or when the Kaplan–Meier estimator did not reach the specified percentile within the study period.

					Percentile Estimates			
Neighbor	Bark Texture	Host Tree	Attach %	*n*	*T*_25_	*T*_50_	*T*_75_	Rate (1/*t*_50_*)*
ghost orchid	smooth	cypress	100	4	23 (-, - ^z^)	23 (-, -)	23 (-, -)	0.04
ghost orchid	semi corrugated	pond apple	100	1	23 (-, -)	23 (-, -)	23 (-, -)	0.04
ghost orchid	corrugated	pop ash	75	4	23 (-, -)	23 (23, -)	- ^y^	0.04
ghost orchid	corrugated	pond apple	100	2	23 (23, -)	29 (23, -)	35 (23, -)	0.03
moss	smooth	cypress	50	2	23 (23, -)	-	-	
moss	semi corrugated	cypress	100	2	23 (-, -)	23 (-, -)	23 (-, -)	0.04
moss	semi corrugated	pop ash	87.5	8	23 (23, 35)	29 (23, 35)	35 (23, -)	0.03
moss	semi corrugated	pond apple	83.3	6	23 (23, 35)	29 (23, -)	47 (23, -)	0.03
moss	corrugated	cypress	75	4	35 (23, -)	-	-	
moss	corrugated	pop ash	75	8	23 (-, -)	23 (-, -)	23 (23, -)	0.04
moss	corrugated	pond apple	100	3	23 (23, -)	23 (23, -)	35 (23, -)	0.04
lichen	smooth	cypress	33	3	23 (23, -)	-	-	0.04
lichen	semi corrugated	cypress	100	2	23 (-, -)	23 (-, -)	23 (-, -)	0.04
lichen	semi corrugated	pop ash	33	3	23 (23, -)	-	-	
lichen	semi corrugated	pond apple	75	4	23 (23, 35)	29 (23, -)	-	0.03
lichen	corrugated	cypress	100	3	23 (23, -)	23 (23, -)	35 (23, -)	0.04
lichen	corrugated	pop ash	56.3	16	23 (23, 35)	35 (23, -)	-	0.03
lichen	corrugated	pond apple	100	3	23 (-, -)	23 (-, -)	23 (-, -)	0.04
bromeliad	smooth	cypress	100	2	23 (-, -)	23 (-, -)	23 (-, -)	0.04
bromeliad	semi corrugated	cypress	0	1	-	-	-	
bromeliad	semi corrugated	pop ash	60	5	23 (23, -)	35 (23, -)	-	0.03
bromeliad	semi corrugated	pond apple	16.7	6	-	-	-	
bromeliad	corrugated	pop ash	44.4	9	23 (23, -)	-	-	0.04
bromeliad	corrugated	pond apple	66.67	3	23 (23, -)	23 (23, -)	-	0.04
fern	semi corrugated	pop ash	100	2	23 (-, -)	23 (-, -)	23 (-, -)	0.04
fern	semi corrugated	pond apple	0	6	-	-	-	
fern	corrugated	cypress	100	1	35 (-, -)	35 (-, -)	35 (-, -)	0.03
fern	corrugated	pop ash	50	6	35 (23, -)	-	-	
fern	corrugated	pond apple	33.3	3	23 (23, -)	-	-	
			69.5 (67.3, 71.6)			26.2 (25.7, 26.6)		0.04 (0.04, 0.04)

^z^ Confidence intervals do not vary from calculated value. Or, upper confidence interval does not vary from lower confidence interval. ^y^ The Kaplan–Meier estimator did not reach a failure probability >0.50 or 0.75.

## Appendix C

**Table A3:** Generalized linear mixed-effects model (GLMM) results for flowering probability of reintroduced *Dendrophylax lindenii*. The model was fit using a binomial error distribution with a logit link function and included linear and quadratic terms of scaled time (month) to evaluate non-linear temporal dynamics in flowering probability. Estimate represents the fixed-effect regression coefficient on the logit scale. SE denotes the standard error of the coefficient. Z Value is the Wald test statistic for each parameter estimate, and *p* indicates the associated two-tailed significance level.

	Estimate	SE	Z Value	*p*
Intercept	−2.7308	1.0282	−2.656	0.007911
Month (linear)	−16.4935	8.1858	−2.015	0.043915
Month (quadratic)	−28.5049	7.9540	−3.584	0.000339
